# Clinical, radiological and molecular characterization of intramedullary astrocytomas

**DOI:** 10.1186/s40478-020-00962-1

**Published:** 2020-08-08

**Authors:** Laetitia Lebrun, Barbara Meléndez, Oriane Blanchard, Nancy De Nève, Claude Van Campenhout, Julie Lelotte, Danielle Balériaux, Matteo Riva, Jacques Brotchi, Michaël Bruneau, Olivier De Witte, Christine Decaestecker, Nicky D’Haene, Isabelle Salmon

**Affiliations:** 1 Department of Pathology, Erasme University Hospital, Université Libre de Bruxelles (ULB), 808 Route de Lennik, B-1070 Brussels, Belgium; 2 Molecular Pathology Research Unit, Department of Pathology, Virgen de la Salud Hospital, Toledo, Spain; 3 Department of Pathology, Cliniques Universitaires Saint-Luc, Université Catholique de Louvain (UCLouvain), Brussels, Belgium; 4 Department of Neuroradiology, Erasme University Hospital, Université Libre de Bruxelles (ULB), Brussels, Belgium; 5 Department of Neurosurgery, Mont-Godinne University Hospital, UcL Namur, Yvoir, Belgium; 6 Department of Neurosurgery, Erasme University Hospital, Université Libre de Bruxelles (ULB), Brussels, Belgium; 7 DIAPath, Center for Microscopy and Molecular Imaging, ULB, Gosselies, Belgium; 8 Laboratory of Image Synthesis and Analysis, Brussels School of Engineering/École Polytechnique de Brussels, ULB, Brussels, Belgium

**Keywords:** Intramedullary astrocytomas-glial tumor-spinal cord-targeted next-generation sequencing-*H3F3A* K27M-*KIAA1549-BRAF*

## Abstract

Intramedullary astrocytomas (IMAs) are rare tumors, and few studies specific to the molecular alterations of IMAs have been performed. Recently, *KIAA1549-BRAF* fusions and the *H3F3A* p.K27M mutation have been described in low-grade (LG) and high-grade (HG) IMAs, respectively. In the present study, we collected clinico-radiological data and performed targeted next-generation sequencing for 61 IMAs (26 grade I pilocytic, 17 grade II diffuse, 3 LG, 3 grade III and 12 grade IV) to identify *KIAA1549-BRAF* fusions and mutations in 33 genes commonly implicated in gliomas and the 1p/19q regions. One hundred seventeen brain astrocytomas were analyzed for comparison. While we did not observe a difference in clinico-radiological features between LG and HG IMAs, we observed significantly different overall survival (OS) and event-free survival (EFS). Multivariate analysis showed that the tumor grade was associated with better OS while EFS was strongly impacted by tumor grade and surgery, with higher rates of disease progression in cases in which only biopsy could be performed. For LG IMAs, EFS was only impacted by surgery and not by grade. The most common mutations found in IMAs involved *TP53, H3F3A* p.K27M and *ATRX*. As in the brain, grade I pilocytic IMAs frequently harbored *KIAA1549-BRAF* fusions but with different fusion types. Non-canonical IDH mutations were observed in only 2 grade II diffuse IMAs. No *EGFR* or *TERT* promoter alterations were found in IDH wild-type grade II diffuse IMAs. These latter tumors seem to have a good prognosis, and only 2 cases underwent anaplastic evolution. All of the HG IMAs presented at least one molecular alteration, with the most frequent one being the *H3F3A* p.K27M mutation. The *H3F3A* p.K27M mutation showed significant associations with OS and EFS after multivariate analysis. This study emphasizes that IMAs have distinct clinico-radiological, natural evolution and molecular landscapes from brain astrocytomas.

## Introduction

The recently updated 4th edition of the World Health Organization (WHO) Classification of Central Nervous System (CNS) tumors in 2016 (WHO 2016) drastically changed histological diagnosis in neuropathology by integrating molecular data into daily diagnostic practice [30, 51]. The current WHO 2016 classification of CNS tumors is based on clinical criteria, histology and molecular characteristics to achieve accurate determination of the prognosis and response to treatment and to improve patient management [30]. The WHO 2016 classification is applied using the same criteria for supra-tentorial, infra-tentorial, posterior fossa and intramedullary gliomas, regardless of location. However, the vast majority of molecular data on gliomas were obtained from studies of tumors localized in the brain [5, 50].Some studies, however, have shown that particular molecular alterations are related to tumor location. Fusions involving the *BRAF* and *KIAA1549* genes are found in nearly 80% of cerebellar grade I astrocytomas but only 50–55% of non-cerebellar grade I cases [24]. Similarly, the p.K27M somatic mutation in the *H3F3A* gene (H3K27M) [41] predominates in high-grade (HG) infiltrative astrocytomas in midline locations, mainly the brain stem, thalamus and spinal cord [44].

Only a few studies specifically addressing intramedullary gliomas are available. Intramedullary tumors are rare CNS neoplasms accounting for 2 to 4% of all CNS tumors [41, 48]. The majority of these (80%) are gliomas, which are ependymomas (60–70%) and astrocytomas (30–40%) [4].

The few available studies specific to the molecular alterations of intramedullary astrocytomas (IMAs) have been performed and have shown that the most frequent recurrent molecular alterations are fusions involving *KIAA1549-BRAF* and H3K27M mutations [41]. To date, very few cases with IDH mutations have been described in IMAs, and these mutations were not classic *IDH1* p.R132H and *IDH2* p.R172H mutations [9, 12, 41, 47, 54].

Nevertheless, while the genetic profiles of brain astrocytomas have been largely established, those of uncommon IMAs remain to be defined. The present study aimed to correlate clinical, radiological and molecular data of IMAs to improve the current knowledge about the prognosis and molecular profile of these tumors.

## Materials and methods

### Patient cohort

Institutional Review Board approval for a retrospective analysis of archival biobank tissue was obtained from Biobanque Hôpital Erasme-ULB (BERA), BE_NBWB1, Biothèque Wallonie Bruxelles (BWB), BBMRI-ERIC) and Biolibrary of Saint-Luc University Hospital, together with ethical agreement. For the present study, the following inclusion criteria were defined: pathological diagnosis of LG or HG astrocytoma and spine location. We excluded cases with a secondary supratentorial location at diagnosis. Pathological diagnosis was reviewed by two neuropathologists (LL and IS) according to the WHO 2016 classification [30]. According to the cIMPACT-NOW (the Consortium to Inform Molecular and Practical Approaches to CNS Tumor Taxonomy) Update 4, the presence of the *KIAA1549-BRAF* fusion gene led to grade I pilocytic astrocytoma diagnosis in cases for which a differential diagnosis between grade I pilocytic and grade II diffuse astrocytomas could not be made [13]. Grade III astrocytoma diagnosis was based on mitotic activity, high cellularity, nuclear atypia without features of glioblastoma (necrosis and microvascular proliferation). To note, for the three grade III astrocytomas, as surgical resection was not complete according to surgical reports, we could not exclude undersampled glioblastoma. The final cohort consisted of 61 patients with IMAs diagnosed between 1989 and 2019 from Erasme and Saint-Luc University Hospital. Twenty-six of the tumors were grade I pilocytic astrocytomas, 17 were grade II diffuse astrocytomas, 3 were grade III astrocytomas and 12 grade IV astrocytomas. Three cases were ruled LG astrocytomas because no diagnosis of grade I or grade II could be made. To compare the molecular profile of this cohort of IMAs with their brain counterparts, we used a set of 117 samples obtained from astrocytoma patients consecutively diagnosed between 2017 and 2019 that were not located in spine and that were analyzed by next-generation sequencing (NGS) in our daily practical routine diagnosis setting using gene-targeted “clinical glioma” and “*KIAA1549-BRAF* fusion” panels (see below). This series included 16 grade I astrocytomas, 13 grade II astrocytomas, 13 grade III astrocytomas and 75 grade IV astrocytomas.

**Fig. 1:**
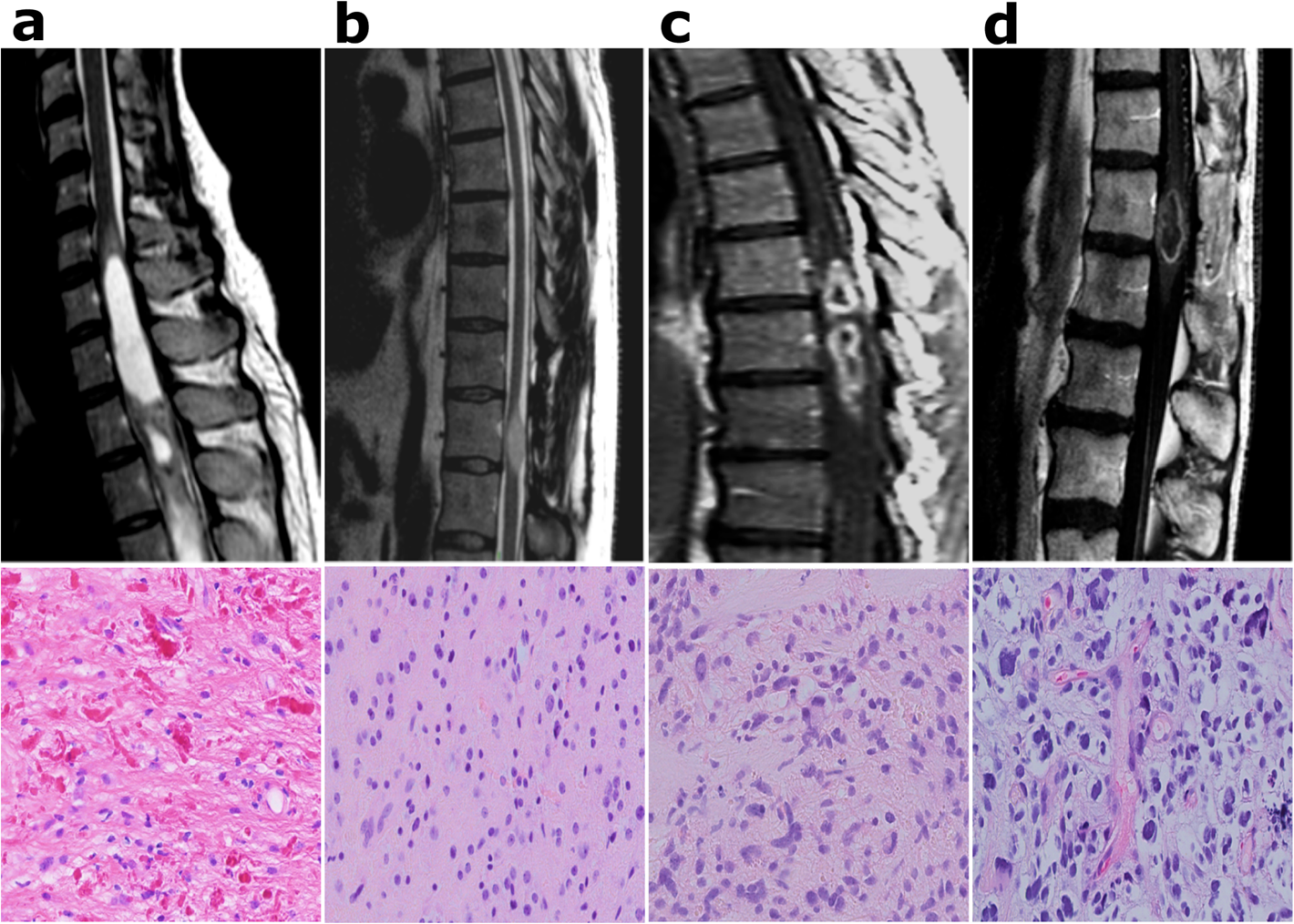
Representative radiological (sagittal MRI images) and histological (HE staining, 200×) features of intramedullary astrocytomas (IMAs) of grade I pilocytic (T2WI) (**a**), grade II diffuse (T2WI) (**b**), grade III (GdT1WI) (**c**) and grade IV (GdT1WI) (**d**) IMAs. *HE*, hematoxylin-eosin; *MRI,* magnetic resonance imaging; *T2WI*, T2 weighted image, *GdT1WI,* gadolinium enhanced T1 weighted images

Magnetic resonance imaging (MRI) exams were reviewed for available cases (*n* = 34) by an expert neuroradiologist (DB). For the remaining cases, imaging features were collected from MRI reports. The imaging features assessed were location, signal T1 and T2 intensity, contrast enhancement, well-delineated vs. infiltrative pattern, cystic component and necrosis. Figure 1 illustrates representative radiological and pathological features of IMAs.

Clinical variables were collected based on clinical and surgical reports: age, gender, extent of surgery (biopsy, partial or total resection), adjuvant treatments, follow-up duration and survival status. Based on MRI reports (contrast enhancement and/or tumor size increase), event-free survival (EFS) was assessed as the time until recurrence after gross total resection or disease progression after biopsy/partial resection, as defined by Pekmezci et al. [34] The median follow-up durations were 4.08 years (range: 0.1 to 27.8) and 1.92 years (range: 0.2 to 4.8) for LG and HG IMAs, respectively. For 54 cases, sufficient material from the primary resection samples was available for molecular analysis. For the remaining 7 cases, we used subsequent surgery samples to perform our molecular tests.

### Next-generation sequencing (NGS) assays

Briefly, DNA and RNA from formalin-fixed paraffin-embedded (FFPE) tumor tissues were extracted using the QIAamp DNA FFPE Tissue Kit (Qiagen, Manchester, UK) and the Maxwell RSC DNA FFPE Kit (Promega Corporation, Madison, WI, USA) and were quantified using a Qubit 2.0 Fluorometer (ThermoFisher Scientific, Waltham, MA, USA). Library construction and quantification were performed using the Ion AmpliSeq Library Kit v2.0 and the Ion Library Quantitation Kit (ThermoFisher Scientific), respectively. Libraries were multiplexed and submitted for emulsion PCR using the Ion Chef System and were sequenced using the Ion GeneStudio S5 System, according to the manufacturer’s instructions (ThermoFisher Scientific).

#### DNA-based targeted NGS

For DNA analysis, two Ampliseq gene-targeted custom panels (“Clinical Glioma” and “Research Glioma” panels) were used to analyze a total of 33 genes commonly implicated in gliomas and the 1p and 19q regions (Supplementary Table S1) [6, 10]. Cases for which the number of mapped reads was < 100,000 and/or the average base coverage was <500x were considered non-informative.

#### RNA-based targeted NGS

To detect *KIAA1549-BRAF* fusions, we used an Ampliseq custom panel that tests 10 different fusions described in the COSMIC database (Sanger Institute Catalogue of Somatic Mutations in Cancer) (Supplementary Table S1). For each RNA sample, more than 20,000 total mapped reads were considered, as recommended by the manufacturer (ThermoFisher Scientific), with at least 5 control genes expressed over 250 reads.

#### Data analyses

For DNA analyses, reads were aligned against the hg19 reference genome with the Torrent Mapping Alignment Program (TMAP, ThermoFisher). After alignment, exonic nonsynonymous and splice variants were filtered to exclude those with fewer than 100 reads, present in the population with a minor allele frequency of more than 1% according to the 1000 Genomes project, with mutant allele coverage of fewer than 30 reads or with mutant allele frequency lower than 5% (except for well-known hotspot mutations). The final list of mutations was visualized with the Integrative Genomic Viewer (IGV, Broad Institute), and systematic sequencing errors or FFPE artifacts were removed. Curated information on variants was obtained from the gnomAD (genome aggregation database, https://gnomad.broadinstitute.org/), COSMIC (http://www.sanger.ac.uk/cosmic) and CbioPortal (https://www.cbioportal.org/) databases. Variants were classified as ones with pathogenic/potential clinical significance or as variants of unknown significance (VUS) following previous recommendations [29].

Ion reporter software (ThermoFisher) was used for copy number variation detection, including high-level amplification of *EGFR* and *PDGFRA* and homozygous deletion of *CDKN2A*. The algorithm uses normalized read coverage across amplicons to predict the copy number or ploidy state. Sample read coverage was compared to a baseline coverage constructed from 10 male control diploid DNA samples. Copy number variation (CNV) data were filtered to exclude regions with low confidence, as recommended by the manufacturer (ThermoFisher).

For RNA analyses, reads were aligned with the hg19 human reference genome, and fusions were identified by using Ion Reporter software v5.10 (ThermoFisher).

### Statistical analysis

Statistical analyses were performed using Statistica software (StatSoft, Tulsa, USA).

After checking the application conditions, the chi-square test or Fisher’s exact test was used to analyze the associations between categorical variables.

Survival data were subjected to Kaplan-Meier analysis and the log-rank test. Multivariate Cox regression method was also applied. Overall survival (OS) and event-free survival (EFS) were calculated from the initial diagnosis. To include categorical variables in the multivariate models, we created dummy variables as follows. The extent of resection was introduced by means of 2 binary variables: biopsy (no/yes) and total resection (no/yes), with partial resection corresponding to the value of 0 for each of these 2 binary variables. In some cases, the total resection variable could be not included because of the problem of singular matrix decomposition for estimating model parameters. Statistical significance was defined as *p* < 0.05.

## Results

### Comparison of clinico-radiological features in low- and high-grade intramedullary astrocytomas

**Fig. 2:**
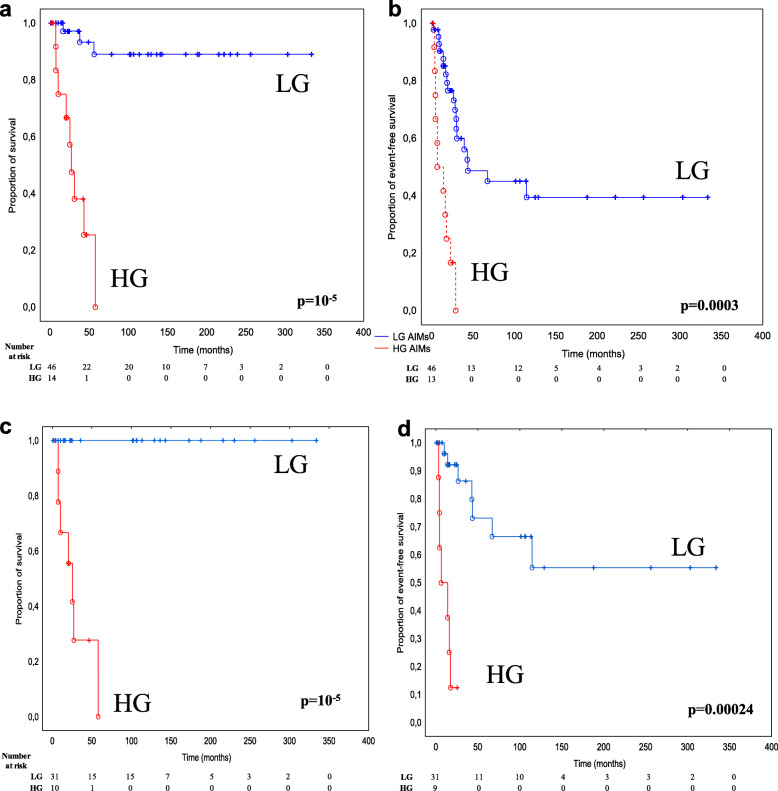
Overall survival (OS) and event-free survival (EFS) of low-grade (LG) and high-grade (HG) intramedullary astrocytomas. Kaplan-Meier curves of OS **(a, c)** and EFS **(b, d)** for all cases **(a, b)** and only for resected (partial and gross total resection) cases **(c, d).***p*-values were calculated using the log-rank test

Table 1 details the radiological and clinical characteristics observed in our series of LG (46 cases) and HG (15 cases) IMA patients. The pediatric population (< 18 years old) consisted of 10 of 46 LG (22%) and 6 of 15 HG (40%) astrocytoma patients. Statistical tests showed that there were no significant differences between LG and HG astrocytomas in terms of age, gender, radiological features and the extension of surgical resection (Fisher test: *p* > 0.05). However, despite these radiological and clinical similarities, OS and EFS differed widely between patients with LG and HG tumors (Fig. 2a-b). HG astrocytoma patients presented an extremely poor prognosis, and no patient survived longer than 50 months (median OS and EFS of 23 and 6 months, respectively). In contrast, LG astrocytoma patients presented very good OS, with 90% of patients surviving after 50 months. Nevertheless, we observed frequent recurrence after gross total resection or disease progression after biopsy/partial resection among the LG IMA patients (median EFS, 67 months), although only 2 LG cases showed progression to a higher grade.

**Table 1:** Clinico-radiological features of 61 low-grade (LG) and high-grade (HG) intramedullary astrocytomas

Variable	Total	LG	HG	Fisher test (except ^1^)
	*n* = 61 (%)	*n* = 46 (%)	*n* = 15 (%)	
**Age** (mean 32, range 4–73 years)				
< 18 years	16 (26)	10 (22)	6 (40)	*p* = 0.188
≥ 18 years	45 (74)	36 (78)	9 (60)	
**Gender**				
Male	32 (52)	24 (52)	8 (53)	*p* = 1.000
Female	29 (48)	22 (45)	7 (47)	
**Tumor location**				
Cervical	18 (30)	14 (30)	4 (27)	NA^1^
Cervico-Thoracic	20 (33)	16 (35)	4 (27)	
Thoracic	17 (28)	12 (26)	5 (33)	
Thoraco-Lumbar	4 (7)	3 (7)	1 (7)	
Lumbar	2 (3)	1 (2)	1 (7)	
**Infiltration**				
Well-delienated	12 (27)	11 (31)	1 (13)	*p* = 0.413
Infiltrative	32 (73)	25 (69)	7 (87)	
No data	17	10	7	
**Contrast enhancement**				
No	11 (18)	9 (20)	2 (13)	
Yes	49 (82)	36 (80)	13 (87)	*p* = 0.713
No data	1	1	0	
**Extent of surgical resection**				
Gross total resection	10 (17)	7 (15)	3 (25)	*p* = 0.149^2^
Partial resection	32 (55)	24 (52)	8 (67)	
Biopsy	16 (28)	15 (33)	1 (8)	
No data	3	0	3	
**Adjuvant treatments**				
No	45 (75)	44 (96)	1 (7)	***p*** **= 10**^**−5**^
Yes	15 (25)	2 (4)	13 (93)	
No data	1	0	1	

^1^Chi-square test; ^2^Comparison of biopsy and surgical resection (gross total and partial); “No data” was excluded from the statistical analyis; *NA* Not applicable

We observed heterogeneity in terms of the extent of surgical resection (Table 1). Gross total resection, partial resection and biopsy were performed in 17, 55 and 28% of patients, respectively, with no statistically significant difference between LG and HG IMAs. Therefore, given this heterogeneity, we applied multivariate Cox survival analysis to assess the potential contribution of surgery (considering the two dummy variables: biopsy and total resection) to OS and EFS, independently of tumor grade. For OS, only the biopsy variable could be added to the model (because of the problem of singular matrix decomposition), and it did not contribute to OS independently of grade (*p* = 0.15), whereas a highly significant contribution of grade was confirmed (*p* = 0.000007, Supplementary Table S2a). In contrast, biopsy is a negative prognostic variable which is independent of tumor grade for EFS (*p* = 0.003), and total surgical resection did not significantly contribute to better prognosis as an independent factor (*p* = 0.408) (Supplementary Table S2b and Fig. 2c-d).

### Comparison of clinico-radiological features in grade I pilocytic and grade II diffuse intramedullary astrocytomas

Analyses of LG IMAs revealed no significant differences between grade I pilocytic and grade II diffuse astrocytomas in terms of age and gender (Table 2). Analyses of LG IMAs revealed no significant differences between grade I pilocytic and grade II diffuse astrocytomas in terms of age and gender (Table 2). We observed that 67% (12/18) of grade I pilocytic astrocytomas showed an infiltrative pattern and that only 33% (6/18) of them were well-delineated. 29% (5/17) grade II diffuse astrocytomas were well-delineated tumors and 71% (12/17) showed an infiltrative pattern. No significant difference was observed between grade I pilocytic and grade II diffuse astrocytomas in terms of infiltration. In contrast, we observed that grade I pilocytic astrocytomas were associated with contrast enhancement (96%, 25/26) significantly more often than grade II diffuse astrocytomas (59%, 10/17) (Fisher test: *p* = 0.004).

**Table 2:** Clinico-radiological features of 46 low-grade (LG) intramedullary astrocytomas

Variable	Grade I	Grade II	LG	Fisher test^**1**^ (except ^2^)
	*n* = 26 (%)	*n* = 17 (%)	*n* = 3 (%)	
**Age (mean 33, range 4–73 years)**				
< 18 years	7 (27)	2 (12)	1 (33)	*p* = 0.211
≥ 18 years	19 (73)	15 (88)	2 (67)	
**Gender**				
male	12 (46)	9 (35)	3 (100)	*p* = 0.451
female	14 (54)	8 (31)	0 (0)	
**Tumor location**				
Cervical	8 (31)	5 (29)	1 (33)	NA^2^
Cervico-Thoracic	10 (38)	5 (29)	1 (33)	
Thoracic	5 (19)	7 (41)	0 (0)	
Thoraco-Lumbar	2 (8)	0 (0)	1 (33)	
Lumbar	1 (4)	0	0	
**Infiltration**				
Well-delienated	6 (33)	5 (29)	0 (0)	*p* = 0.546
Infiltrative	12 (67)	12 (71)	1 (100)	
No data	8	0	2	
**Contrast enhancement**				
No	1 (4)	7 (41)	1 (50)	
Yes	25 (96)	10 (59)	1 (50)	***p*** **= 0.004**
No data	0	0	1	
**Extent of surgical resection**				
Gross total resection	5 (19)	2 (12)	0 (0)	***p*** **= 0.011**^**3**^
Partial resection	17 (65)	6 (35)	1 (33)	
Biopsy	4 (15)	9 (53)	2 (67)	
**Adjuvant treatments**				
No	24 (92)	17 (100)	3 (100)	*p* = 0.359
Yes	2 (8)	0	0	

^1^The three LG cases for which grade I or grade II diagnosis could not be made and category “No data” were excluded from the statistical analyses; ^2^chi-square test; ^3^comparison of biopsy and surgical resection (gross total and partial); *NA* Not applicable

We observed a significant difference in terms of the proportion of patients who received surgery (total or partial resection) between grade I pilocytic and grade II diffuse astrocytomas (84%, 22/26 vs. 47%, 8/17; Fisher test: *p* = 0.011) (Table 2).

**Fig. 3:**
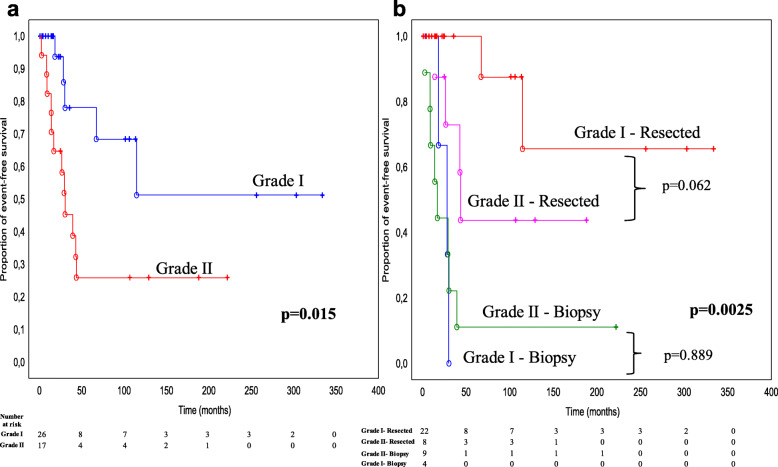
Event-free survival (EFS) of grade I pilocytic and II diffuse intramedullary astrocytomas. Kaplan-Meier curves of EFS for grade I pilocytic and grade II diffuse IMAs without **(a)** and after **(b)** stratification using the extent of surgical resection: biopsy vs. (partially or totally) resected cases. p-values were calculated using the log-rank test

EFS was longer in grade I pilocytic astrocytomas (median EFS, 115 months) than in grade II diffuse astrocytomas (median EFS, 30 months) (log-rank test: *p*-value = 0.015) (Fig. 3a). We did not observe significant differences in EFS (*p* = 0.21, data not shown) for pediatric vs. adult LG patients.

Among LG tumors, surgery strongly impacted EFS in both grade I pilocytic and grade II diffuse IMAs (log-rank test: *p*-value = 0.0025, Fig. 3b). As shown in Fig. 3b, there were no statistically significant differences in terms of EFS between grade I and II patients, either in biopsy or in resected cases (log-rank tests: *p* = 0.889 and *p* = 0.062, respectively). However, without distinguishing between grade I and II, there was a statistically significant difference in EFS between biopsy and resected LG cases (*p* = 0.0008, data not shown).

Multivariate Cox regression combining grade (I vs. II) and two surgery variables (biopsy and total resection) revealed that only the absence of surgery (i.e., biopsy) significantly and negatively impacted the EFS of LG astrocytomas, independently of the other variables in the model (Supplementary Table S3). Additional analyses showed that none of the other clinico-radiological variables (listed in Table 2) contributed to the prognosis independent of the biopsy variable (data not shown).

### Molecular profiles of intramedullary astrocytomas

**Fig. 4:**
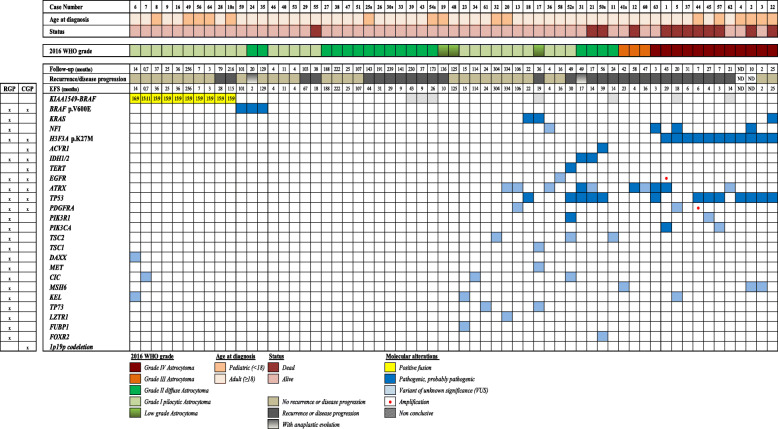
Molecular characterization of 61 intramedullary astrocytomas (IMAs) obtained by targeted next-generation-sequencing using research, clinical and *KIAA1549-BRAF* fusion panels. The figure summarizes the clinico-pathological features and molecular alterations found in the 61 IMAs. *EFS*, event-free survival; *ND*, no data available; *s*, second surgery sample; *RGP*, research glioma panel; *CGP*, clinical glioma panel​

The molecular findings are summarized in Fig. 4. The most common mutations found in the cohort of IMAs studied here concerned *TP53, H3F3A* p.K27M and *ATRX* in 21% (13/61), 18% (11/61) and 13% (10/61) of cases, respectively. Pathogenic hotspot mutations were identified: *BRAF* p.V600E mutation (5%, 3/61), *KRAS* p.G12V/S and p.Q61R (5%, 3/61), *IDH1* p.R132S (2%, 1/61), *IDH2* p.R172M (2%, 1/61), *ACVR1* p.G328V (2%, 1/61) and *TERT* promoter (2%, 1/61). Other variants were identified in the *NF1, PDFGRA, TSC2, TSC1, MSH6, CIC, KEL, PIK3CA, PIK3R1, EGFR, TP73, LZTR1, DAXX, MET, FUBP1* and *FOXR2* genes, most of them being VUS. In contrast to brain astrocytomas, no hotspot *IDH1* p.R132H or *IDH2* p.R172H mutations were identified, and none of our cases harbored the *1p19q* codeletion. Regarding the *KIAA1549-BRAF* fusion, 16% of the contributive cases (10/54) were positive. The different breakpoints identified were *KIAA1549(15)-BRAF(9)* (8 cases), *KIAA1549(16)-BRAF(9*) (1 case) and *KIAA1549(15)-BRAF(11)* (1 case).

Alterations in genes involved in the MAP kinase signaling pathway were frequently found in LG IMAs (39%, 16/41 informative cases): *BRAF* alterations (mutations and fusions), *KRAS* and *NF1* mutations were identified in 13, 2 and 1 cases, respectively. For grade I pilocytic IMAs, we observed MAP kinase signaling pathway alterations in 50% of cases (13/26). For the remaining grade I pilocytic IMAs, we did not detect any molecular alteration in 5 cases, while 8 cases presented at least one variant, including mutations of *EGFR* (one case), *PDGFRA* (one case), *PIK3R1* and the *TERT* promoter (one case) (Fig. 4). Alterations in genes involved in the MAP kinase signaling pathway were found in 15% (2/13) of grade II diffuse IMAs, three had mutations in *IDH* (2 cases) and *ACVR1* (1 case), and 8 grade II diffuse IMAs did not have any alterations identified with the gene-targeted strategy used here.

All HG IMA cases presented at least one mutation. The most frequent one was the *H3F3A* p.K27M mutation, which was found in 73% of cases (11/15) including 11 of 12 grade IV IMAs. Four of the HG IMAs did not harbor the *H3F3A* p.K27M mutation. Remarkably, the only grade IV IMA that did not carry this mutation was diagnosed in a Lynch syndrome context (case 63) and the others HG IMAs were grade III IMAs. The only alteration identified in 2 of these 3 grade III IMAs cases was the mutation of *ATRX. ATRX* mutations were identified in only 2 of 11 of the H3K27M-mutant cases (18%), while 3 of 4 HG H3K27M-wild-type IMAs harbored *ATRX* alterations.

Another common molecular alteration in HG IMAs was *TP53* mutation (53%, 8/15), which was identified in most H3K27M-mutant tumors (64%, 7/11). In addition, the majority of *NF1* mutations (3 of 4), as well as the only *PIK3CA* mutation identified, were found in HG IMAs. Importantly, none of the HG cases harbored *KIAA1549-BRAF* fusions and/or *BRAF* p.V600E mutations.

### Molecular profile comparison of intramedullary and brain astrocytomas

We compared the molecular profiles of IMAs described above to those of their brain counterparts using a series of gliomas analyzed in the routine diagnostic setting of our laboratory. Examination of the gene-targeted “Clinical Glioma” and “*KIAA1549-BRAF* fusion” panels revealed that 98.2% (112/114) of brain astrocytomas had at least one molecular alteration (mutation or fusion), while only 65% (36/55) of IMAs had at least one alteration (Fisher test: *p* < 10^− 6^, Table 3).

**Table 3:** Molecular profile of brain (*n* = 117) and intramedullary astrocytomas (IMAs) (*n* = 58; the 3 low-grade IMAs cases were removed) obtained with the Clinical Glioma Panel (CGP) and *KIAA1549-BRAF* fusion panel

	Brain			Spine		
	***n*** = 117 (%)	Molecular alterations types, n		***n*** = 58 (%)	Molecular alterations types, n	
**Grade I**	16 (14)	Tumors with at least one molecular alteration : 14/16		26 (45)	Tumors with at least one molecular alteration : 17/24^1^	
		. ***KIAA1549-BRAF***: 14/16 Fusions type: *9 KIAA1549 (16) BRAF (9), 2 KIAA1549 (15) BRAF (9),* *2 KIAA1549 (16) BRAF (11), 1 KIAA1549 (13) BRAF (11)*			. ***KIAA1549-BRAF***: 10/26 Fusions type: *8 KIAA1549 (15) BRAF (9), 1 KIAA1549 (16) BRAF (9)* *1 KIAA1549 (15)-BRAF (11)*	
		No *ATRX, TP53, PDGFRA, BRAF, EGFR, TERT* molecular alterations			Others:	
					. *ATRX* mutation: 3/24	. *BRAF* p.V600E mutation: 1/24
					. *TP53* mutation: 2/24	. *EGFR* mutation: 1/24
					. *PDFGRA* mutation: 1/24	. *TERT* promoter mutation: 1/24
**Grade II**	13 (11)	Tumors with at least one molecular alteration : 13/13		17 (29)	Tumors with at least one molecular alteration : 5/16^1^	
		**IDH-mutated (*n*= 7): All*IDH1*p.R132H mutations** . *TP53* mutation: 7/7 . *ATRX* mutation: 5/7 . *TERT* promoter mutation: 1/7			**IDH-mutated (*n*= 2):*IDH1*p.R132S and*IDH2*p.R172M mutations** *. TP53* mutation : 2/2 *. ATRX* mutation: 2/2	
		**IDH-wt (*n*= 6)**: . *TERT* promoter mutation: 4/6 . *EGFR* mutation: 1/6, Amplification: 1/6 . *ATRX* mutation: 1/6 . *PTEN* mutation: 1/6 . *KIAA1549(14) -BRAF(9):* 1/6 (Pilomyxoid Astrocytoma)			**IDH-wt (*n*= 14)** . *BRAF* p.V600E mutation: 2/14 . *TP 53* mutation : 1/14 . *ACVR1* mutation: 1/14	
		No *BRAF* p.V600E, *TP53, ACVR1* molecular alterations			No *TERT, EGFR, ATRX, PTEN* molecular alterations	
**Grade III**	13 (11)	Tumors with at least one molecular alteration : 13/13		3 (5)	Tumors with at least one molecular alteration : 2/3	
		**IDH-mutated (*n*= 9):*IDH1*p.R132H(*n*= 5), p.R132C(*n*= 2), p.R132S(*n*= 1), p.R132G(*n*= 1)** . *TP53* mutation: 9/9 . *ATRX* mutation: 6/9 . *CDKN2A* mutation: 1/9 . *PDGFRA* mutation: 1/9			*. ATRX* mutation: 2/3	
		**IDH-wt (*n*= 4)** . *TERT* promoter mutation: 3/6 . *EGFR* amplification: 2/6 . *EGFR* mutation: 1/6 . *ATRX* mutation: 1/6				
**Grade IV**	75 (64)	Tumors with at least one molecular alteration : 72/72^1^		12 (21)	Tumors with at least one molecular alteration : 12/12	
		**IDH-mutated (*n*= 5):*IDH1*p.R132H (*n*= 4), p.R132C (*n*= 1)** . *TP53* mutation: 5/5 . *ATRX* mutation: 2/5 . *PTEN* mutation: 1/5			No IDH mutations	
		**IDH-wt (*n*= 67)**			**IDH-wt (*n*= 12)**	
		*. TERT* promoter mutation: 57/67			. *H3F3A* mutation: 11/12	. *PDGFRA* molecular alterations: 2/12
		. *EGFR* amplification: 20/67	. *EGFR* mutation: 16/67		. *TP53* mutation: 8/12	. *EGFR* amplification: 1/12
		. *TP53* mutation: 24/67	. *H3F3A* mutation: 1/67		. *ATRX* mutation: 3/12	
		. *PTEN* mutation: 18/67	. *BRAF* p.V600E mutation: 1/67		No *TERT*promoter, *PTEN*, *CDKN2A*, *BRAF* molecular alterations	
		. *ATRX* mutation: 4/67	. *PDGFRA* molecular alteration: 5/67			
		. *CDKN2A* molecular alteration: 3/67				

^1^The difference in the number of total cases is due to non-informative cases for the CGP (2 grade I pilocytic, 1 grade II diffuse IMAs and 3 brain grade IV astrocytomas); *wt* wild-type

At least one molecular alteration was found in 87.5% (14/16) and 71% (17/24) of brain and spinal grade I pilocytic astrocytoma cases, respectively (Fisher test: *p* = 0.27) with the *KIAA1549-BRAF* fusion being the most common molecular alteration found in both locations. Nevertheless, although the *KIAA1549(16)-BRAF(9)* fusion was most common in brain grade I pilocytic astrocytomas (64.3%, 9/14), the *KIAA1549(15)-BRAF(9)* fusion was the most common in spinal grade I pilocytic astrocytomas (80%, 8/10 Fisher test: *p* = 0.65). No other mutations were found in brain grade I pilocytic astrocytomas, while 21% (5/24) of grade I pilocytic IMAs presented mutations in the *TP53* or *ATRX* gene (Fisher test: *p* = 0.071).

One hundred percent (13/13) of the brain grade II diffuse astrocytomas harbored at least one molecular alteration, compared to only 31.3% (5/16) in the spine (Fisher test: *p* = 0.00013). *IDH1* p.R132H was the most common mutation found in brain grade II diffuse astrocytomas (54%, 7/13), while only 2 spine grade II diffuse astrocytomas cases harbored *IDH* mutations (12.5%, 2/16; Fisher test: *p* = 0.041), and these mutations were non-canonical IDH mutations. In both brain and spine tumors, *TP53* and *ATRX* mutations were found concomitantly with IDH mutations. Among IDH-wild-type grade II diffuse astrocytomas, the *TERT* promoter mutation was the most common mutation found in the brain (67%, 4/6) compared to *BRAF* p.V600E (14%, 2/14) mutation in the spine (Fisher test: *p* = 0.037).

For HG astrocytomas, no comparison between grade III astrocytomas of the brain and spine was possible because of the small number of samples from the spine. However, all grade IV astrocytomas, either in the brain or in the spine, harbored at least one mutation. *TERT* promoter mutation was the most common molecular alteration found in grade IV astrocytomas of the brain (79%, 57/72), while the *H3F3A* p.K27M mutation was found in nearly all grade IV IMAs (92%, 11/12). Moreover, no IDH mutations were found in grade IV astrocytomas located in the spine, while 6.9% (5/72) of the grade IV astrocytomas were mutated in the brain (Fisher test: *p* = 1.0). Among IDH-wild-type grade IV brain astrocytomas, *EGFR* alterations (mutation and amplification) and *TP53* and *PTEN* mutations were the most common molecular alterations after *TERT* promoter mutation. However, in spine grade IV astrocytomas, *TP53* and *ATRX* mutations were the most common ones after *H3F3A* p.K27M mutation, and no *PTEN* and *TERT* promoter mutations were found.

### Prognostic implications of molecular alterations in intramedullary astrocytomas

Among the 11 H3K27M-mutant IMA cases, 8 of 11 cases died, with a median OS of 20 months, and 7 of 11 showed recurrence after gross total resection or disease progression after biopsy/partial resection with a median EFS of 6 months. In contrast, *KIAA1549-BRAF* fusion was associated with good prognosis; none of the *KIAA1549-BRAF*-positive cases died, and only 2 of them had recurrence after gross total resection or disease progression after biopsy/partial resection after delays of 28 (case 28) and 115 months (case 10s).

Among grade I pilocytic IMAs (*n* = 26), the absence of the *KIAA1549-BRAF* fusion (*n* = 16) did not contribute significantly to the prognosis in terms of EFS (log rank test: *p* = 0.690, data not shown).

Of the 3 LG IMAs cases which died, two harbored *TP53* mutations; one of these two cases harbored non-canonical (non-*IDH1* p. R132H and non-*IDH2* p.R172H) IDH mutation (*IDH2* p.R172M) (case 21) and the other, an *ACVR1* p.G328V mutation (case 50). For the third case (case 55), no molecular alterations could be identified. In the two LG IMAs cases that progressed to a higher grade, one harbored a non-canonical IDH mutation (*IDH1* p.R132S) (case 31) and the other a *BRAF* p.V600E mutation (case 24).

Among HG IMAs (*n* = 15), H3K27M-wild-type cases (*n* = 4) seemed to have a better prognosis; only 1 case died (median follow-up of 29 months) compared to 8 cases among H3K27M-mutant cases (*n* = 11) (median follow-up of 20 months). Statistical analyses comparing H3K27M-mutant and H3K27M-wild-type HG IMAs could not be performed due to the small number of H3K27M-wild-type cases.

We then investigated whether molecular alterations significantly impacted patients’ survival independently of the surgical treatment. The OS-related results detailed in Supplementary Table S4a showed that only the H3K27M mutation made a significant contribution as a poor prognostic factor independent of the other variables in the model. Regarding EFS, (Supplementary Table S4b) only the biopsy and the H3K27M mutation variables contributed significantly and negatively to prognosis, independently of the other variables in the model. For EFS in grade I pilocytic and grade II diffuse IMAs (see Supplementary Table S5), no significant contribution of the molecular group indicators was found.

## Discussion

Integration of some clinico-radiological criteria with histology is commonly performed for the diagnosis of brain gliomas [20, 49]. In the present study, we observed that these criteria were not helpful for IMAs. In brain gliomas, contrast enhancement is generally a common feature of HG gliomas [36, 49], although it is a non-specific finding and can also be found in grade I pilocytic astrocytoma [31, 33, 40, 49]. In our study, we did not observe a difference in contrast enhancement between LG and HG IMAs. This could be explained by the fact that 96% of grade I pilocytic IMAs included in our series showed contrast enhancement. Interestingly, similar infiltrative patterns were observed in both grade I pilocytic and grade II diffuse IMAs, while grade I pilocytic and grade II diffuse brain astrocytomas are, by definition, well-circumscribed and diffuse neoplasms, respectively. Nevertheless, in the spine, assessing the infiltrative/diffuse pattern by MRI can sometimes be challenging [2]. Our data are thus consistent with the literature and suggest that these classical radiological features are not helpful in the differential diagnosis between LG and HG IMAs.

Nevertheless, even if the histological grade can be challenging to assess in the spine [32, 41], it remains the most powerful prognostic marker [21, 52], with LG IMAs associated with better outcomes than HG IMAs [11, 21, 55]. Our study showed that the tumor grade was associated with better OS, while EFS was strongly impacted by tumor grade and surgery, with a higher rate of disease progression in cases in which only biopsy could be performed. In the literature, the impact of surgery on outcomes remains highly debated [16, 39]. It is interesting to note that although the univariate analysis showed that the EFS was longer in grade I pilocytic IMAs than in grade II diffuse IMAs, the multivariate analysis showed that when the type of surgery was taken into account, the distinction between grade I and II did not add significant prognostic value. This result was explained by the fact that, within each group of patients treated with the same type of surgery, the difference in EFS between grades I and II was not statistically significant. This result is consistent with Diaz et al., who did not find grade to be an independent prognostic factor among LG IMAs [11], and Karikari et al.*,* who nearly reported the same rate of recurrence between grade I (41.7% recurrence) and grade II (40% recurrence) [26]. This outcome strongly contrasts with brain location, for which most grade I pilocytic astrocytomas are classically associated with better outcomes.

IMAs also appear to be molecularly distinct from brain astrocytomas. Grade I pilocytic astrocytomas were characterized by *KIAA1549-BRAF* fusions in both locations. However, the most frequent breakpoints found in IMAs were different than those usually found in brain grade I pilocytic astrocytomas, as also described by Faulkner et al. [14]. The impact of the presence of the *KIAA1549-BRAF* fusion on prognosis remains debated [14], with some studies showing associations with better outcomes for pediatric LG gliomas [22, 24]. In our study, *KIAA1549-BRAF* had no impact on either EFS or OS independent of surgery when considering all cases nor on EFS when considering only grade I pilocytic IMAs.

Molecular alterations of grade II diffuse astrocytomas differ according to their location. IDH mutations were found in half of the brain astrocytoma cases, but only in 2 of the 61 IMAs (with non-canonical mutations). IDH mutations are generally rare and mostly non-canonical in midline locations [35]. Previous reports of non-canonical IDH mutations in the spine are scarce and restricted to very few cases [8, 9, 12, 41, 54] with Takai et al. reporting one case with the same *IDH1* p.R132S mutation that we found [47]. While for brain gliomas, IDH mutations are commonly associated with good prognosis, in our cohort of LG IMAs, the EFS of the two IDH-mutated cases was 17 and 14 months while for IDH-wild-type cases, the median EFS was 67 months. Among the 2 IDH-mutated cases, one died, and one progressed to a higher grade, as also reported by Takai et al., who described a short OS (11 months) for an *IDH1* p.R132S-mutated grade II spinal cord astrocytoma [47]. This finding could suggest that rare IDH mutations could be related to more aggressive behavior in LG IMAs, but this hypothesis must be confirmed in a larger cohort.

As described recently, “adult-type” grade II diffuse astrocytomas with no IDH mutations often harbor *EGFR* amplification*, TERT* promoter mutations or combined whole chromosome 7 gain and whole chromosome 10 loss and have glioblastoma-like behavior [3]. In contrast, “pediatric-type” grade II diffuse astrocytomas, rarely exhibiting IDH mutations, were recently described to more frequently have “non-R132H *IDH1*” mutations in pediatric hemispheric diffuse astrocytomas [46], and they are associated with an indolent clinical behavior [13]. The molecular alterations of these “pediatric-type” grade II diffuse astrocytomas were recently described and concerned the *BRAF* p.V600E mutation, *FGFR1* alterations or *MYB* or *MYBL1* rearrangement [13, 37]. In our cohort of grade II diffuse IMAs, we did not observe *EGFR* or *TERT* promoter mutations. We observed *BRAF* p.V600E in 2 cases and found no other specific molecular alteration except for one hotspot *ACVR1* p. G328V mutation, mostly described in “diffuse intrinsic pontine glioma” (DIPG) [23] and in one case of pediatric spinal glioblastoma in the literature [28]. However, *FGFR1*, *MYB* and *MYBL1* were not covered by the gene-targeted NGS used in the present study. Recent studies have reported that chromosomal rearrangements in gliomas resulting in transcript fusion gene, such as *NTRK 1/2/3* or *FGFR1/2/3* fusions, could be therapeutic targets [15] and are implicated in clinical trials (Entrectinib [15] or Larotrectinib in pediatric patients [19]). Clinical trials involving *FGFR* inhibitors showed promising effects as targeted treatments for gliomas [15, 18]. Because of their potentially clinical implications, it could be interesting to test these fusion genes.

In the brain, the natural evolution of most “adult-type” grade II diffuse gliomas is to progress to a higher grade [11, 37]. Anaplastic evolution occurred in only two grade II diffuse IMAs in this cohort, in agreement with previous studies that reported only one of fifteen grade II diffuse [39] and six of thirteen grade III IMAs progressing to a higher grade [38]. This outcome could suggest a unique biological behavior of IMAs, and it raises the question of whether or not anaplastic evolution and therefore secondary grades III and IV astrocytomas in the spine occur, as rarely observed in “pediatric-type” LG gliomas [37].

In this cohort, nearly all grade IV IMAs were H3K27M mutants (92%), in line with previous reports, which described a range between 38 and 100% of H3K27M-mutant tumors among HG spinal astrocytomas [1, 8, 17, 25, 27, 32, 35, 41, 42, 44, 53]. All of the *H3F3A* mutations identified in our cohort consisted of the recurrent hotspot p.K27M mutation. We did not find any other mutations in genes encoding histone variants H3.1 or additional *H3F3A* mutations, in contrast with the recent report by Sloan et al.*,* who reported *H3F3A* p.G34W in grade II and III spinal cord astrocytomas [43].We did not find *TERT* promoter mutations among HG IMAs, in contrast with those in the brain, and with two recent studies [1, 8]. Alvi et al. described distinct prognostic groups of HG IMAs according to the presence of *TERT* promoter mutations and H3K27M mutations [1]. The median OS of H3K27M-mutant IMAs in our cohort was 21 months, while Alvi et al. and Yi et al. reported a median OS of 48 months and 40 months, respectively [1, 53]. These results could suggest better OS for H3K27M-mutant IMAs than in other midline locations, for which the median OS is 12 months or less [7, 45]. Interestingly, none of the three grade III IMAs studied here showed H3K27M mutations or *TERT* promoter mutations, and the only alteration identified was the *ATRX* mutation in two of them.

No recurrent molecular alterations other than *KIAA1549-BRAF* fusion and *H3F3A* p.K27M mutations were identified in our cohort, in agreement with Shankar et al. [41] and the recent report of Zhang et al.*,* who did not report any recurrent molecular alterations [54].

## Conclusions

In conclusion, these specific clinico-radiological and molecular landscapes of IMAs suggest that diagnostic algorithms commonly used in the brain must be reviewed to be confirmed as appropriate for those in the spine. First, assessment of the imaging infiltrative/well-delineated pattern, as commonly used for the differential diagnosis of grade I pilocytic astrocytomas and diffuse grade II, III and IV astrocytomas, seems not to be useful in the spine. Moreover, classical molecular alterations such as IDH mutations, *EGFR* and *TERT* promoter mutations, associated with diagnosis and prognosis in the brain, do not seem to occur with the same frequency, have the same implications in spine or at least, deserve further study. In the brain, assessment of IDH mutations has been shown, as Ellezam et al. reported, to help to distinguish grade I pilocytic, typically IDH wild-type, from grade II diffuse astrocytomas [12]. In the spine, because of the rarity of IDH mutations, the absence of IDH mutations cannot help to distinguish grade I pilocytic astrocytoma from grade II diffuse astrocytoma. Moreover, IDH immunohistochemistry would not be useful because of the absence of classical *IDH1* p.R132H in IMAs.

In addition, the prognostic implications of molecular alterations in IMAs must be well characterized since implications similar to those observed in the brain cannot be established. LG IMAs seem to have a clinical and a molecular profile more related to diffuse “pediatric-type” gliomas than those classically observed in the brain. HG IMAs are strongly related to diffuse midline H3K27M mutant gliomas, and the *H3F3A* p.K27M mutation is an important molecular alteration to assess because of its prognostic implications.

## Supplementary information

**Additional file 1: Table S1.** List of genes, copy number variations (CNV) and *KIAA1549-BRAF* fusions tested.

**Additional file 2: Table S2.** Prognosis Model for all cases.

**Additional file 3: Table S3.** Event-Free Survival (EFS) Prognosis Model for LG cases (grade I and grade II only).

**Additional file 4: Table S4.** Prognosis Model including molecular data for all cases.

**Additional file 5: Table S5.** Event-free Survival (EFS) Prognosis Model including molecular data for grade I pilocytic and grade II diffuse intramedullary astrocytomas.

## Data Availability

The datasets used and/or analyzed during the current study are available from the corresponding author upon reasonable request.

## Abbreviations

CNS: Central Nervous System
CGP: Clinical Glioma Panel
CNV: Copy Number Variations
*H3F3A* p.K27M: H3K27M
HG: High-Grade
IMAs: Intramedullary Astrocytomas
LG: Low-Grade
MRI: Magnetic Resonance Imaging
NGS: Next Generation Sequencing
OS: Overall Survival
EFS: Event-Free Survival
RGP: Research Glioma Panel
VUS: Variants of Unknown Significance
WHO: World Health Organization
