# Supplementary material for: Clinical, radiological and molecular characterization of intramedullary astrocytomas
Source: Acta Neuropathol Commun. 2020 Aug 8;8:128. doi: 10.1186/s40478-020-00962-1 (PMC7414698; doi:10.1186/s40478-020-00962-1)
Supplement: Supplementary file 1 — Additional file 1: Table S1. List of genes, copy number variations (CNV) and KIAA1549-BRAF fusions tested. [file 40478_2020_962_MOESM1_ESM.pdf]

**Supplementary Table S1.** List of genes, copy number variations (CNV) and *KIAA1549-BRAF* fusions tested

| DNA-based panels | | | | | |
|---|---|---|---|---|---|
| **Gene** | **RefSeq** | **Research panel (30-gene panel)[1]** | | **Clinical panel (16-gene panel)[2]** | |
| | | **Target** | **Overall Coverage (%)** | **Target** | **CNV targeted** |
| ***ACVR1*** | NM_001105 | - | - | Hotspots in exons 6, 7, 8, 9 | - |
| ***ATRX*** | NM_000489 | Whole coding sequence, exons 1-35 | 95,9 | Whole coding sequence, exons 1-35 | - |
| ***BRAF*** | NM_004333 | Whole coding sequence, exons 1-18 | 94,6 | Hotspots in exons 7, 10, 11, 15 | - |
| ***CDKN2A*** | NM_000077 | Whole coding sequence, exons 1-3 | 50,9 | Whole coding sequence, exons 1-3 | Homozygous deletion |
| ***CDKN2B*** | NM_004936 | Whole coding sequence, exons 1-2 | 64,7 | - | - |
| ***CIC*** | NM_015125 | Whole coding sequence, exons 1-20 | 90,4 | - | - |
| ***DAXX*** | NM_001141970 | Whole coding sequence, exons 1-8 | 90,1 | - | - |
| ***EGFR*** | NM_005228 | Whole coding sequence, exons 1-28 | 99,9 | Whole coding sequence, exons 1-28 | Amplification |
| ***FOXR2*** | NM_198451 | Whole coding sequence, exon 1 | 100,0 | - | - |
| ***FUBP1*** | NM_003902 | Whole coding sequence, exons 1-20 | 98,8 | - | - |
| ***H3F3A*** | NM_002107 | Whole coding sequence, exons 2-3 | 63,7 | Hotspot in exon 2 | - |
| ***HIST1H3B*** | NM_003537 | Whole coding sequence, exon 1 | 95,2 | Hotspot in exon 1 | - |
| ***HIST1H3C*** | NM_003531 | Whole coding sequence, exon 1 | - | Hotspot in exon 1 | - |
| ***IDH1*** | NM_005896 | Whole coding sequence, exons 3-10 | 97,7 | Hotspot in exon 4 | - |
| ***IDH2*** | NM_002168.2 | Whole coding sequence, exons 2-11 | 90,8 | Hotspot in exon 4 | - |
| ***KEL*** | NM_000420 | Whole coding sequence, exons 1-19 | 99,3 | - | - |
| ***KRAS*** | NM_033360 | Whole coding sequence, exons 2-6 | 90,3 | - | - |
| ***LZTR1*** | NM_006767 | Whole coding sequence, exons 1-21 | 96,2 | - | - |
| ***MET*** | NM_001127500 | Whole coding sequence, exons 2-21 | 98,9 | - | - |
| ***MSH6*** | NM_000179 | Whole coding sequence, exons 1-10 | 94,8 | - | - |
| ***NF1*** | NM_001042492 | Whole coding sequence, exons 1-58 | 98,5 | - | - |
| ***NOP53*** | NM_015710 | Whole coding sequence, exons 1-13 | 72,0 | - | - |
| ***PDGFRA*** | NM_006206 | Whole coding sequence, exons 2-23 | 100,0 | Exons 5-12, 14-15, 18, 21-23 | Amplification |
| ***PIK3CA*** | NM_006218 | Whole coding sequence, exons 2-21 | 96,9 | - | - |
| ***PIK3R1*** | NM_181523 | Whole coding sequence, exons 2-16 | 98,4 | - | - |
| ***PTEN*** | NM_000314 | Whole coding sequence, exons 1-9 | 95,7 | Whole coding sequence, exons 1-9 | - |
| ***QKI*** | NM_006775 | Whole coding sequence, exons 1-8 | 98,6 | - | - |
| ***RB1*** | NM_000321 | Whole coding sequence, exons 1-27 | 93,1 | - | - |
| ***TERT*** | NM_001193376 | - | - | Hotspot in promoter region | - |
| ***TP53*** | NM_000546 | Whole coding sequence, exons 2-11 | 100,0 | Whole coding sequence, exons 2-11 | - |
| ***TP73*** | NM_005427 | Whole coding sequence, exons 2-14 | 86,0 | - | - |
| ***TSC1*** | NM_000368 | Whole coding sequence, exons 3-23 | 94,8 | - | - |
| ***TSC2*** | NM_000548 | Whole coding sequence, exons 2-42 | 96,8 | - | - |
| ***Chr 1p*** | - | - | - | - | Codeletion |
| ***Chr19q*** | - | - | - | - | Codeletion |

| RNA-based panel | |
|---|---|
| **Fusion** | **COSMIC** |
| *KIAA1549(19) - BRAF(9)* | COSF511 |
| *KIAA1549(18) - BRAF(10)* | COSF509 |
| *KIAA1549(17) - BRAF(10)* | COSF509 |
| *KIAA1549(16) - BRAF(10)* | COSF1283 |
| *KIAA1549(16) - BRAF(11)* | COSF485 |
| *KIAA1549(16) - BRAF(9)* | COSF481 |
| *KIAA1549(17) - BRAF(10)* | COSF1284 |
| *KIAA1549(15) - BRAF(11)* | COSF1226 |
| *KIAA1549(15) - BRAF(9)* | COSF483 |
| *KIAA1549(13) - BRAF(9)* | COSF1474 |

[1] Cantero D, Rodríguez de Lope Á, Moreno de la Presa R, Sepúlveda JM, Borrás JM, Castresana JS, D'Haene N, García JF, Salmon I, Mollejo M, Rey JA, Hernández-Laín A, Meléndez B (2018) Molecular Study of Long-Term Survivors of Glioblastoma by Gene-Targeted Next-Generation Sequencing. J Neuropathol Exp Neurol 77:710–716. doi: 10.1093/jnen/nly048

[2] D'Haene N, Meléndez B, Blanchard O, De Nève N, Lebrun L, Van Campenhout C, Salmon I (2019) Design and Validation of a Gene-Targeted, Next-Generation Sequencing Panel for Routine Diagnosis in Gliomas. Cancers (Basel) 11. doi: 10.3390/cancers11060773
