# Supplementary material for: Clinical, radiological and molecular characterization of intramedullary astrocytomas
Source: Acta Neuropathol Commun. 2020 Aug 8;8:128. doi: 10.1186/s40478-020-00962-1 (PMC7414698; doi:10.1186/s40478-020-00962-1)
Supplement: Supplementary file 2 — Additional file 2: Table S2. Prognosis Model for all cases. [file 40478_2020_962_MOESM2_ESM.pdf]

Supplementary Table S2: Prognosis Model for all cases

Multivariate analysis for Overall Survival (OS) and Event-Free Survival (EFS)

Variables		Hazard Ratio[1]	95% CI	p-value
**a. OS**	High Grade (no/yes)	29,6	(6.75-129.870)	**0,000007**
	Biopsy (no/yes)	2,67	(0.693-10.023)	0,155
**b. EFS**	High Grade (no/yes)	14,06	(4.715-41.916)	**0,000002**
	Biopsy (no/yes)	3,76	(1.559-9.044)	**0,003**
	Total Resection (no/yes)	0,53	(0.116-2.403)	0,408

[1]for "yes" category. NB: The total resection variable could not be included for the OS because of problem of singular matrix decomposition for estimating model parameters.
