# Supplementary material for: Clinical, radiological and molecular characterization of intramedullary astrocytomas
Source: Acta Neuropathol Commun. 2020 Aug 8;8:128. doi: 10.1186/s40478-020-00962-1 (PMC7414698; doi:10.1186/s40478-020-00962-1)
Supplement: Supplementary file 3 — Additional file 3: Table S3. Event-Free Survival (EFS) Prognosis Model for LG cases (grade I and grade II only). [file 40478_2020_962_MOESM3_ESM.pdf]

Supplementary Table S3 : Event-Free Survival (EFS) Prognosis Model for LG cases (grade I and grade II only)

Multivariate analysis of EFS			
Variables	Hazard Ratio[1]	95% CI	p-value
Grade II (no/yes)	1,7	(0.534-5.390)	0,37
Biopsy (no/yes)	4,94	(1.501-16.223)	**0,009**
Total Resection (no/yes)	0,42	(0.049-3.603)	0,429

[1]for "yes" category
