# Supplementary material for: Clinical, radiological and molecular characterization of intramedullary astrocytomas
Source: Acta Neuropathol Commun. 2020 Aug 8;8:128. doi: 10.1186/s40478-020-00962-1 (PMC7414698; doi:10.1186/s40478-020-00962-1)
Supplement: Supplementary file 4 — Additional file 4: Table S4. Prognosis Model including molecular data for all cases. [file 40478_2020_962_MOESM4_ESM.pdf]

Supplementary Table S4: Prognosis Model including molecular data for all cases

Multivariate analysis for Overall Survival (OS) and Event-Free Survival (EFS)				
	Variables	Hazard Ratio[1]	95% CI	p-value
**a. OS**	Biopsy (no/yes)	1,28	(0.34-4.85)	0,72
	H3K27M mutation (no/yes)	72,78	(8.61-615.12)	**0,000083**
**b. EFS**	Biopsy (no/yes)	3,69	(1.46-9.32)	**0,0058**
	KIAA1549-BRAF fusions (no/yes)	0,83	(0.18-3.80)	0,8075
	H3K27M mutation (no/yes)	10,52	(2.76-40.05)	**0,0006**

[1]for "yes" category
