# Supplementary material for: Clinical, radiological and molecular characterization of intramedullary astrocytomas
Source: Acta Neuropathol Commun. 2020 Aug 8;8:128. doi: 10.1186/s40478-020-00962-1 (PMC7414698; doi:10.1186/s40478-020-00962-1)
Supplement: Supplementary file 5 — Additional file 5: Table S5. Event-free Survival (EFS) Prognosis Model including molecular data for grade I pilocytic and grade II diffuse intramedullary astrocytomas. [file 40478_2020_962_MOESM5_ESM.pdf]

Supplementary Table S5: Event-free Survival (EFS) Prognosis Model including molecular data for grade I pilocytic and grade II diffuse intramedullary astrocytomas

Multivariate analysis of EFS			
Variables	Hazard Ratio[1]	95% CI	p-value
Biopsy (no/yes)	15,31	(3.74-62.73)	**0,0002**
KIAA1549-BRAF fusions (no/yes)	1,55	(0.31-7.81)	0,593

[1]for "yes" category
